# T_1_–T_2_ Dual-modal MRI contrast agents based on superparamagnetic iron oxide nanoparticles with surface attached gadolinium complexes

**DOI:** 10.1007/s11051-014-2678-6

**Published:** 2014-10-11

**Authors:** Agnieszka Szpak, Sylwia Fiejdasz, Witold Prendota, Tomasz Strączek, Czesław Kapusta, Janusz Szmyd, Maria Nowakowska, Szczepan Zapotoczny

**Affiliations:** 1 Faculty of Chemistry, Jagiellonian University, Ingardena 3, 30-060 Krakow, Poland; 2 Department of Solid State Physics, Faculty of Physics and Applied Computer Science, AGH University of Science and Technology, Mickiewicza 30, 30-059 Krakow, Poland; 3 Faculty of Energy and Fuels, AGH University of Science and Technology, Mickiewicza 30, 30-059 Krakow, Poland

**Keywords:** SPION, Superparamagnetic nanoparticles, Gadolinium, Chitosan, Magnetic resonance imaging, Relaxivity, Composite nanoparticles

## Abstract

Dual-mode MRI contrast agents consisting of superparamagnetic iron oxide nanoparticle (SPION) cores and gadolinium ions associated with the ionic chitosan protecting layer were synthesized and studied. Gadolinium ions were introduced into the coating layer via direct complex formation on the nanoparticles surface, covalent attachment or electrostatically driven deposition of the preformed Gd complex. The modified SPIONs having hydrodynamic diameters ca. 100 nm form stable, well-defined dispersions in water and have excellent magnetic properties. Physiochemical properties of those new materials were characterized using e.g., FTIR spectroscopy, dynamic light scattering, X-ray fluorescence, TEM, and vibrating sample magnetometry. They behave as superparamagnetics and shorten both T_1_ and T_2_ proton relaxation times, thus influencing both r_1_ and r_2_ relaxivity values that reach 53.7 and 375.5 mM^−1^ s^−1^, respectively, at 15 MHz. The obtained materials can be considered as highly effective contrast agents for low-field MRI, particularly useful at permanent magnet-based scanners.

## Introduction

Magnetic resonance imaging (MRI) is recognized as powerful and sensitive diagnostic technique widely used in biomedical field (Gupta and Gupta 2005; Laurent et al. 2008). It is a preferred tool since it does not use ionizing radiation thus allowing to avoid harmful side effects (Stephen et al. 2011). The method is based on magnetic relaxation processes of water protons that are recorded during the scan (Figuerola et al. 2010). It allows for noninvasive body imaging with high spatial resolution which is highly desired in modern clinical diagnostics (De et al. 2011). MRI is frequently used for tumor detection as it provides information on cellular level. Despite the wide range of advantages offered by MR imaging, there are still many challenges to be faced for more accurate diagnosis like accounting for the presence of various artifacts (air bubbles, calcification) or limited sensitivity (Yoo et al. 2011). Application of contrast agents allows to eliminate some of these problems. They play an important role by enhancing the contrast between healthy and diseased tissue by increasing the image quality therefore increasing sensitivity of the method (Cho et al. 2010; Niu et al. 2013). Generally, there are two different classes of MR contrast agents. One type is T_1_ contrast agents that are reducing proton longitudinal relaxation time and providing positive contrast (bright signal) and the second are T_2_ agents that can shorten proton transverse relaxation time causing negative contrast (dark signal) (Santra et al. 2012). Positive contrast agents are typically of paramagnetic nature usually gadolinium complexes or manganese ions, while superparamagnetic materials, mainly these based on iron oxide particles, act as the negative contrast agents (Yanga et al. 2011).

Nanostructural materials have been shown to have some advantages over conventional MRI agents. Nanometer dimensions of these materials have considerable impact on certain parameters like unique magnetic properties or ability to operate on cellular level (Stephen et al. 2011). Recently, superparamagnetic iron oxide nanoparticles (SPIONs), negative contrast agents, have been receiving a great interest among wide range of magnetic nanoparticles studied (Figuerola et al. 2010). In our previous report, we presented synthesis of SPION coated with ultrathin layer of biocompatible ionic polymers in aqueous medium and their physicochemical characterization (Szpak et al. 2013). The obtained results, especially the high value of the r_2_ relaxivity, equal to 369 ± 3 mM^−1^ s^−1^, indicate that these agents can be potentially useful in MR imaging.

In modern diagnosis where highly accurate information is desired, single mode contrast agents are not always sufficient (De et al. 2011). Thus, there is a growing interest in developing multimodal imaging probes (Zhou et al. 2012). Dual-mode T_1_–T_2_ contrast agents, combining the advantages of positive and negative contrasts, may allow for improved diagnosis by sharpening anatomical details in the MR image (De et al. 2011).

However, development of dual-mode agents with strong T_1_–T_2_ contrast effects is very challenging. The problem arises when T_1_ and T_2_ agents are in close proximity (De et al. 2011). For instance, when T_1_ and T_2_ agents are in direct contact there is an interference between them that leads to perturbation in the relaxation process of the paramagnetic T_1_ contrast agent. As a result, an undesired effect of decrease in T_1_-dependant signal is observed (Cho et al. 2010). On the other hand, easy access of water molecules to both materials that influence the relaxation of protons is also crucial.

There are several attempts for creation contrasts with T_1_–T_2_ dual modality reported in literature (Im et al. 2013). Among them, a few approaches seem to be particularly interesting. In the report concerning magnetoliposomes (iron oxide cores in phospholipid bilayer) Gd^3+^ ions were conjugated to the inner and outer bilayer shells by creating appropriate complex (De Cuyper et al. 2007). Seo et al. (2006) presented core–shell type agent with FeCo core and single graphite shell. The r_2_ value was 6 times higher than that for commercially available used contrast agent Ferridex. Bimodal agents prepared by conjugating T_1_ type agent to the surface of magnetic T_2_ structure were also reported (Bae et al. 2010). In the work of Choi et al., silica layer of different thicknesses was used to separate T_1_(Gd_2_O(CO_3_)_2_) and T_2_(MnFe_2_O_4_) contrast modes (Cho et al. 2010). Depending on the thickness of SiO_2_ layer, significant changes in T_1_ were observed.

In this report, we propose three different approaches for preparation of contrast agents with T_1_–T_2_ dual modality. The presented methods have several advantages over those already reported. The obtained SPIONs have the biocompatible coatings that are synthesized in an aqueous medium and the fabrication process is easy and fast. The effect of the obtained agents on the proton T_1_ and T_2_ NMR relaxation times has been investigated and the obtained results are very promising for biomedical applications of the novel contrast agents.

## Materials and methods

### Materials

Iron(III) chloride hexahydrate and iron(II) chloride tetrahydrate (Sigma), ammonia (25 % solution, puriss. p.a) (Sigma), Diethylenetriaminepentaacetic acid (DTPA, for complexometry >99.0 %, Fluka), *N*-Hydroxysuccinimide (NHS, 98 %, Aldrich), Gadolinium(III) chloride hexahydrate 99.999 % (Aldrich), *N,N,N,N*-Tetramethylethylenediamine >99 % (TEMED, BioReagent/SIGMA), *N*-(3-Dimethylaminopropylethylcarbodiimide hydrochloride crystalline (EDC, Sigma-Aldrich), Diethylenetriaminepentaacetic acid gadolinium(III) dihydrogen salt hydrate 97 % (GdDTPA, Aldrich), FeREX^TM^ 10 mg Fe/mL, Ocean NanoTech LCC iron oxide nanocrystals (Ocean) 5 mg/mL, chitosan (low molecular weight, Aldrich). Deionized water was used for preparation of all the solutions.

### Preparation of contrast agents based on SPIONs

#### Synthesis of SPIONs

Cationic (CCh) and anionic (ACh) derivatives of low molecular weight chitosan were obtained according to the procedures described previously (Bulwan et al. 2009). For CCh, the degree of substitution with GTMAC (DS_GTMAC_) estimated by NMR was found to be 57 % and deacetylation degree (%DD) 78 %. For ACh, the degree of substitution with sulfonic groups (DS_TMST_) was found to be 67 %.

**Scheme 1:**
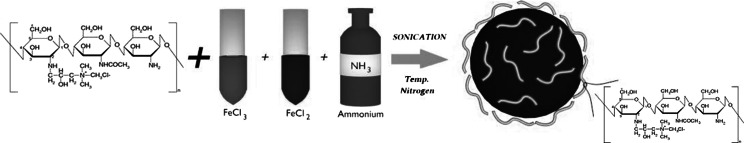
Schematic illustration of the synthetic pathway for obtaining SPION-CCh

SPIONs coated with cationic derivative of chitosan (SPION-CCh) were obtained using the procedure developed by us and described earlier (Szpak et al. 2013). Briefly, iron salts in molar ratio Fe(III):Fe(II) = 2:1, (0.1622 g FeCl_3_·6H_2_O and 0.0596 g FeCl_2_·4H_2_O) were dissolved in 50 mL of aqueous solution of CCh (1 g/L). The solution was deoxygenated by purging with argon and sonicated for 10 min in a thermostated bath (20 °C). Next, 5 mL of 5 M NH_3_(aq) was added drop-wise, and the solution was further sonicated for 30 min (see Scheme 1). Finally, the precipitated nanoparticles, SPION-CCh, were purified by magnetic filtration.

#### Synthesis of contrasts with dual modality SPION-Gd

**Scheme 2:**
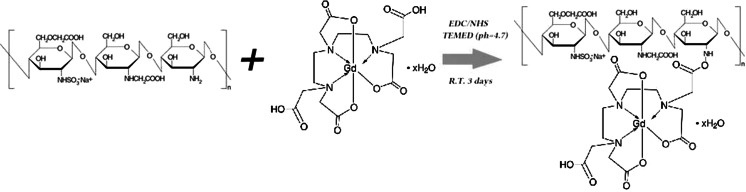
Schematic illustration of the modification of ACh by the Gd complex (GdDTPA)

Three different pathways were proposed to prepare dual-mode contrast agents. Method 1 (GdDTPA). In the first step, ACh polymer was modified with gadolinium complex (see Scheme 2) using slightly modified method that was previously described (Huang et al. 2008). Briefly, 40 mL of 0.1 % ACh in 0.1 M acetic acid was prepared and 100 mg NHS and 250 mg EDC were then added. The pH of the solution was adjusted to 6 by addition of 1 M NaOH solution. Then, 10 mL of 100 mM GdDTPA aqueous solution was added and the mixture was stirred for 24 h at room temperature. The resulting mixture was dialyzed against distilled water for 7 days (water was replaced twice a day) and the modified polymer (ACh-GdDTPA) was lyophilized. In the next step, SPIONs coated with ACh-GdDTPA were prepared. The procedure was as follows: 7.5 mL of ACh-GdDTPA solution (2 g/L) was mixed with 15 mL of purified SPION-CCh dispersion (~50 ug/mL) and sonicated for 10 min. The product was purified by magnetic filtration. As a result, SPION-Gd (ACh-GdDTPA) was obtained.

Method 2 (electrostatic). Firstly, 15 mL of SPION-CCh solution (~50 ug/mL) was mixed with 5 mL of the gadolinium complex (GdDTPA) aqueous solution (21 g/L) and sonicated. After 10 min, 10 mL of ACh solution (2 g/L) was added and again sonicated (10 min). Subsequently, the magnetic filtration was applied. As a result, SPION-Gd (electrostatic) was obtained.

**Scheme 3:**
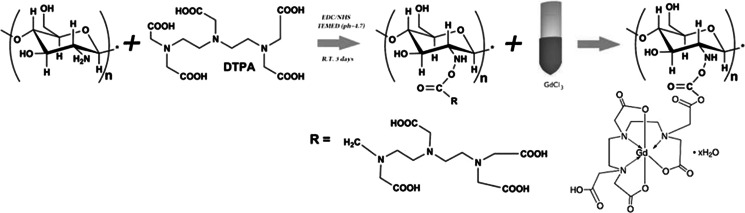
Schematic illustration of the modification of chitosan by DTPA and subsequent formation of complex with Gd^3+^

Method 3 (GdCl_3_). In the first step, DTPA was attached to chitosan macromolecules resulting in formation ChDTPA following the method reported by (Darras et al. 2010). Coupling of DTPA with the low molecular weight chitosan was performed with EDC/NHS as coupling agents. Chitosan (0.5 g) was dissolved in water acidified by addition of HCl (pH = 4.7). Next DTPA (38 mg) was activated with NHS and EDS in TEMED and added to the chitosan solution. The obtained solution was left for 72 h. The product was subsequently dialysed. The expected molar ratio of DTPA grafted to the chitosan for the applied conditions is 10 % (Darras et al. 2010). Next, gadolinium chloride was used in order to create complex with DTPA (see Scheme 3). For that purpose, 10 ml of aqueous solution of chitosan modified with DTPA (ChDTPA) (0.1 g/L) was mixed with 10 mL of 0.2 mM GdCl_3_ water solution. The formed complex was dialyzed against distilled water with 10 kDa membrane for 7 days. The obtained chitosan modified with Gd complex, ChDTPAGd, was used for the synthesis of SPIONs according to the method described for SPION-CCh (see point 2.2.1) but instead of cationic derivative of chitosan (CCh), the ChDTPAGd was used. The SPION-Gd (GdCl_3_) as final product was obtained.

### Apparatus

#### Infrared spectrometry (FTIR)

Fourier transform infrared spectrometry was carried out using Thermo Fisher Scientific Nicolet IR200 spectrometer equipped with ATR accessory.

#### Dynamic light scattering (DLS)

Hydrodynamic sizes and zeta potentials of the obtained nanoparticles were measured by dynamic light scattering (DLS) using ZetaSizer Nano ZS (Malvern Instruments Ltd), equipped with He–Ne laser operating at 633 nm. The measurements were performed at 25 °C, triple for each sample. The mean weighted size according to distribution by volume and by number, as well as, zeta potential was determined.

#### Transmission electron microscopy (TEM)

The size and shape of the selected bimodal agent were characterized by TEM (Tecnai G2 F20 (200 kV) with field emission gun (FEG)). The bright field and high resolution electron microscopy (HREM) images were obtained. SPIONs were sonicated for 2 min before deposition on carbon film and air-dried at room temperature.

#### X-ray fluorescence (XRF)

XRF measurements have been carried out using SEA 1000A analyzer (SII NanoTechnology Inc.) equipped with a rhodium X-ray tube applying bulk analysis mode for dried samples of nanoparticle materials. The calibration of the response (sensitivity) for the individual elements in this mode of analysis is performed by the authorized service based on the respective calibration samples. Thus, there is no need to repeat calibration for each individual measurement. The applied calibration procedure guarantees good enough accuracy for determination of Fe and Gd content in dry samples.

#### VSM

Determination of the magnetic properties of suspensions of nanoparticles was done with a vibrating sample magnetometer option of a quantum design physical property measurement system (PPMS) equipped with a superconducting 9 Tesla magnet. Hysteresis loops have been measured at selected temperatures in the range 3–350 K and magnetic field ranging from −8 to +8 Tesla.

#### NMR

Proton nuclear magnetic resonance (NMR) relaxation times and the line widths were measured with an NMR spin-echo spectrometer-relaxometer PS-15 operating at the frequency of 15 MHz. For the measurements of T_1_ relaxation time, a saturation-recovery sequence was applied and for the T_2_ determination, a Carr-Purcell-Meiboom-Gill sequence was used. For the resonance line measurements, a Fourier transformation of free induction decay after a single π/2 pulse was used. Aqueous dispersions of the nanoparticles at concentration range 1–90 ppm were investigated by applying 5 min sonication prior each measurement.

## Results and discussion

### Preparation of dual-mode agents

**Scheme 4:**
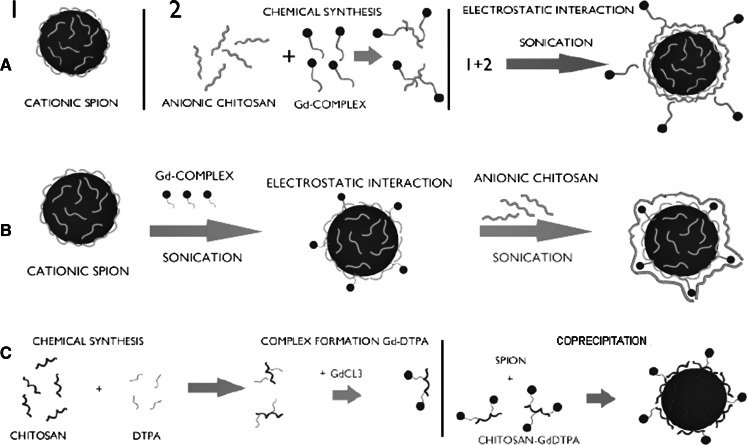
Schematic illustration of the synthetic pathways of bimodal agents: **a** SPION-Gd (ACh-GdDTPA), **b** SPION-Gd (electrostatic), **c** SPION-Gd (GdCl_3_)

As presented at the schematic diagram (Scheme 4), three types of dual-mode contrast agents were prepared based on the method described previously by us for chitosan-coated SPIONs (Szpak et al. 2013). The positively charged SPIONs were formed during one-pot synthesis (co-precipitation method) carried out in an aqueous medium using iron salts (FeCl_3_ and FeCl_2_) and biocompatible polymer, chitosan (CCh), upon addition of ammonia. According to DLS measurements, the obtained nanoparticles form small aggregates with hydrodynamic diameters of about 100 nm. However, the sizes of the magnetic cores were about 12 nm as revealed previously by TEM analysis (Szpak et al. 2013).

These nanoparticles were chosen for further modifications owing to their excellent properties e.g., stability of dispersion in aqueous media, biocompatibility of the coatings, and superior magnetic parameters (high values of magnetic saturation and transverse relaxivity). The possibilities for surface modifications were also of considerable importance. Thus, SPIONs underwent further modification in order to create T_1_–T_2_ bimodal agents, which were carried out in three separate pathways.

The first modification method, yielding final material marked as GdDTPA, involves conjugation of DTPA complex of gadolinium to anionic derivative of chitosan leading to ACh-GdDTPA. Such modified polymer was used to coat the previously prepared SPION-CCh nanoparticles using “layer-by-layer” (LbL) method that involves electrostatic interactions between oppositely charged polyelectrolytes. In the obtained agent, the positive and negative contrast materials are separated by two polymer layers. The actual distance between the iron oxide core and GdDTPA is difficult to be precisely determined but based on the thickness of the CCh/ACh bilayer it may be estimated to be about 2.5 nm (Bulwan et al. 2009).

The second approach is mainly based on electrostatic interactions. Previously prepared positively charged SPION-CCh was mixed with the negatively charged gadolinium complex, (Tan and Zhang 2005), forming SPION-Gd nanohybrid under sonication. Sonication prevents weak physical adsorption (driven by the van der Waals interactions) of the gadolinium complex, that otherwise might be leaking from the coating over the time. To ensure the stability of the obtained agents they were additionally coated with anionic derivative of chitosan, ACh, using LbL. In that material, the superparamagnetic core is separated from the paramagnetic gadolinium ions by cationic chitosan surface layer only with the thickness of about 1.25 nm (Bulwan et al. 2009).

In the third undertaken pathway, the chemical synthesis was carried out. Firstly, DTPA was conjugated with the native chitosan in order to enable complex formation when the polymer was subsequently treated with gadolinium chloride. Then, the synthesis of SPION-Gd nanoparticles using chitosan pre-modified with GdDTPA as protecting component was carried out.

### Physicochemical characterization of single and bimodal agents based on SPION

#### FTIR analysis

**Fig. 1:**
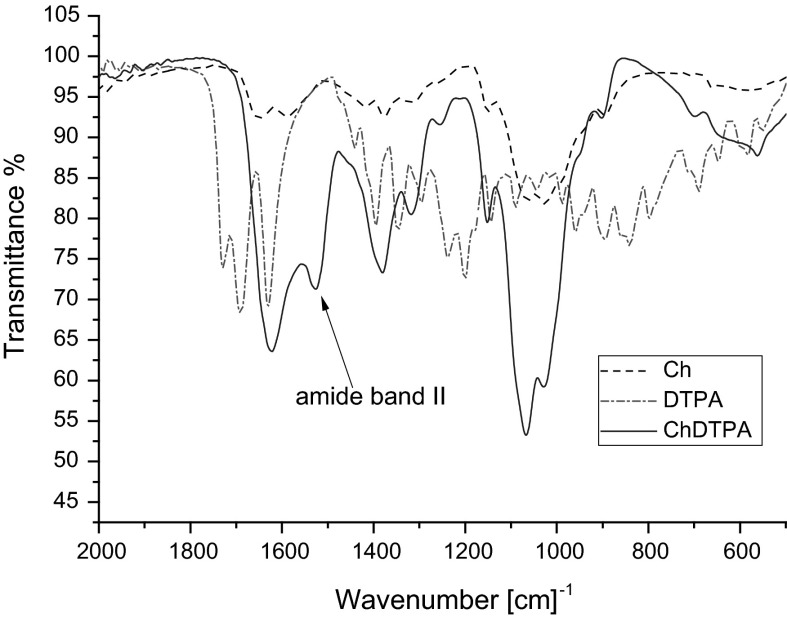
FTIR spectra of : chitosan, DTPA, ChDTPA

In order to check whether DTPA was successfully conjugated with chitosan macromolecules, the infrared spectroscopy was applied. Figure 1 presents FTIR spectra of chitosan, DTPA, and ChDTPA. The IR spectrum of chitosan reveals the broad band between 1,100 and 1,000 cm^−1^ that can be attributed to vibration of C–O bond and to –C–O–C– vibration in the monosaccharide unit, reported also by other authors (Justi et al. 2005). In case of DTPA, the bands at 1,730, 1,696, and 1,630 cm^−1^, corresponding to the C=O bending in –COOH being in the form of monomer, dimer, and carboxylate, respectively, can be noticed. When analyzing ChDTPA sample, the band between 1,100 and 1,000 cm^−1^, characteristic for chitosan, can be observed as well as the band at 1,620 cm^−1^ (C=O group) present in DTPA spectrum. The band at 1,530 cm^−1^ that can be assign to the amide II band, confirms that DTPA was grafted by the amide bond to chitosan in ChDTPA.

**Fig. 2:**
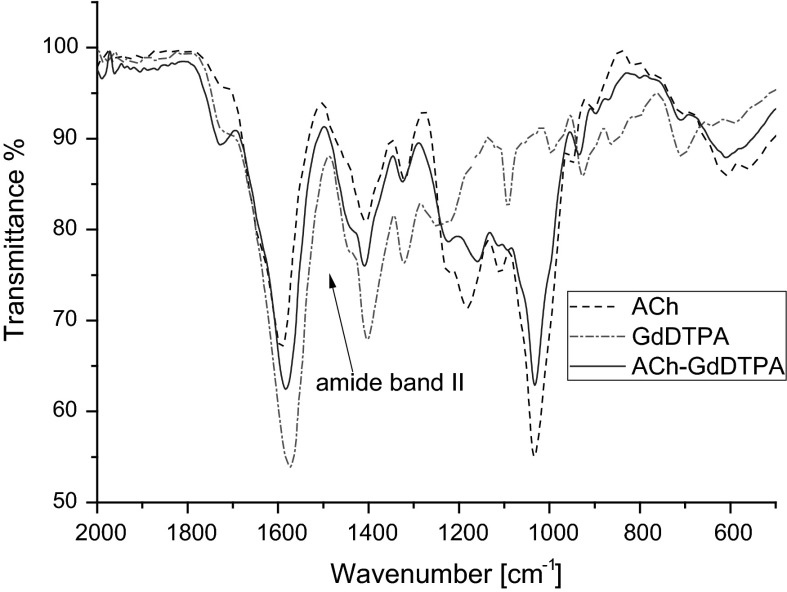
FTIR spectra of: anionic derivative of chitosan ACh, GdDTPA, ACh-GdDTPA

Anionic derivative of chitosan ACh, gadolinium complex GdDTPA, and ACh-GdDTPA were also analyzed by FTIR spectroscopy in order to confirm grafting of GdDTPA to ACh. The spectra of ACh, GdDTPA, and ACh-GdDTPA are presented in Fig. 2. As it can be observed, most of the bands that are characteristic for GdDTPA and for ACh are overlapping. The significant difference in the spectra of ACh and ACh-GdDTPA that can be noticed is broadening of the band corresponding to C=O bond which is caused by overlapping of the amide band. This observation also confirms covalent attachment of GdDTPA to ACh.

#### DLS and zeta potential measurements

DLS and zeta potential measurements were carried out to determine the size and zeta potential values of SPION-based agents obtained in different synthesis pathways. The values of the mean hydrodynamic diameters and the zeta potentials are presented in Table 1. It may be noticed that the hydrodynamic size of the nanoparticles does not change significantly after various treatments and is kept around 100 nm. The zeta potential sign is consistent with the charge of the outer polymeric layer.

**Table 1:** The values of the mean hydrodynamic diameter and zeta potential as measured by DLS for the obtained SPIONs suspended in water

Sample name	Mean diameter (by number) d (nm)	Mean diameter (by volume) d (nm)	Zeta potential ξ (mV)^a^
SPION-Gd (electrostatic)	86	92	−30 ± 5
SPION-Gd (ACh-GdDTPA)	93	94	−34 ± 7
SPION-Gd (GdCl_3_)	134	140	−39 ± 7
SPION-CCh	121	160	+32 ± 6

^a^Average value and its standard deviation

Unfortunately, this is not the case for the method SPION-Gd (GdCl_3_) that should produce positively charged nanoparticles. It is also likely that the formed SPIONs are not fully coated by ChDTPA as indicated by the negative zeta potential (see Table 1) characteristic for the naked SPIONs (Boguslavsky and Margel 2008). What is more, this synthetic method at the applied conditions resulted in unsatisfactory content of Gd (see Table 2) in the material and was excluded from further consideration.

**Table 2:** The content of gadolinium in the obtained agents, given as weight-percent (wt%) or molar-percent mol% with respect to the sum of Fe and Gd present in the sample

Sample name	Gd wt%	Gd mol%
SPION-Gd (electrostatic)	3.8 ± 1.8	1.3 ± 0.6
SPION-Gd (ACh-GdDTPA)	11.5 ± 1.3	4.4 ± 0.5
SPION-Gd (GdCl_3_)	0	0
SPION-CCh	0	0

It is also worth mentioning that all the obtained nanoparticles possess zeta potential values in the range required for formation of stable dispersions. What is more, we have checked the stability of the suspensions of the nanoparticles after 3 months of their storage both: in water and 5 % glucose isotonic solution that is commonly used for injections. For SPION-Gd (ACh-GdDTPA), the measured zeta potential practically did not change from the initial value (see Table 1) indicating high stability of the suspension in both media. These observations are very important for the possible practical applications of the obtained nanoparticles as contrast agents in MRI. Suspension of SPION-Gd (electrostatic) seems to be somehow less stable for long-term storage as the zeta potentials values increased from initial −30 ± 5 mV to −11 ± 3 in the glucose solution and −10 ± 9 in water. That can be explained considering the partial desorption of negatively charged GdDTPA and/or ACh from the coated nanoparticles over the time of storage. These observations additionally confirm the importance of covalent linking of the Gd complex to the polymeric coating of the nanoparticles for their long-term stability in suspension.

Additionally, the effectiveness of the electrostatic coating of SPION-CCh with the gadolinium complex and then ACh was also tested using DLS technique by measuring the zeta potentials. The obtained results show that during the electrostatic coating with GdDTPA the zeta potential of the starting SPION-CCh (**ξ** **=** **+**32 mV) decreases slightly due to the presence of carboxylic groups from the Gd complex but it is still positive (**ξ** **=** **+**13 mV). After adsorption of ACh, the surface charge of the nanoparticles reverses (**ξ** **=** −30 mV) as expected for ACh polymer layer covering the adsorbed Gd complex layer.

**Fig. 3:**
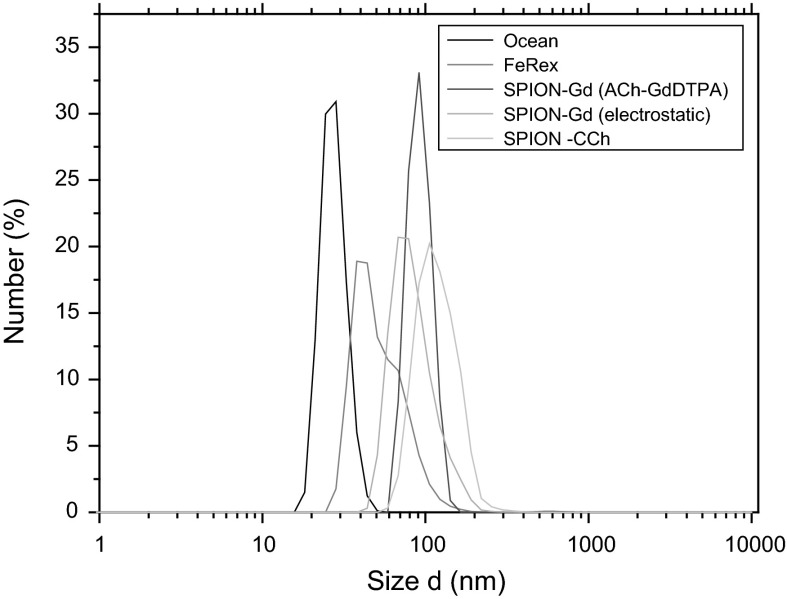
Distribution of hydrodynamic diameters for SPION-CCh, SPION-Gd (ACh-GdDTPA), SPION-Gd (electrostatic) and for commercial products: Iron Oxide from Ocean NanoTech and FeREX^TM^ from BioPAL

The obtained hydrodynamic sizes for the smallest nanoparticles (SPION-Gd (ACh-GdDTPA), SPION-Gd (electrostatic)) were compared with these for the two commercially available products: iron oxide from Ocean NanoTech and FeREX^TM^ from BioPAL (Fig. 3). The hydrodynamic sizes of all nanoparticles are in the same range so the other properties of these objects can be also compared.

**Fig. 4:**
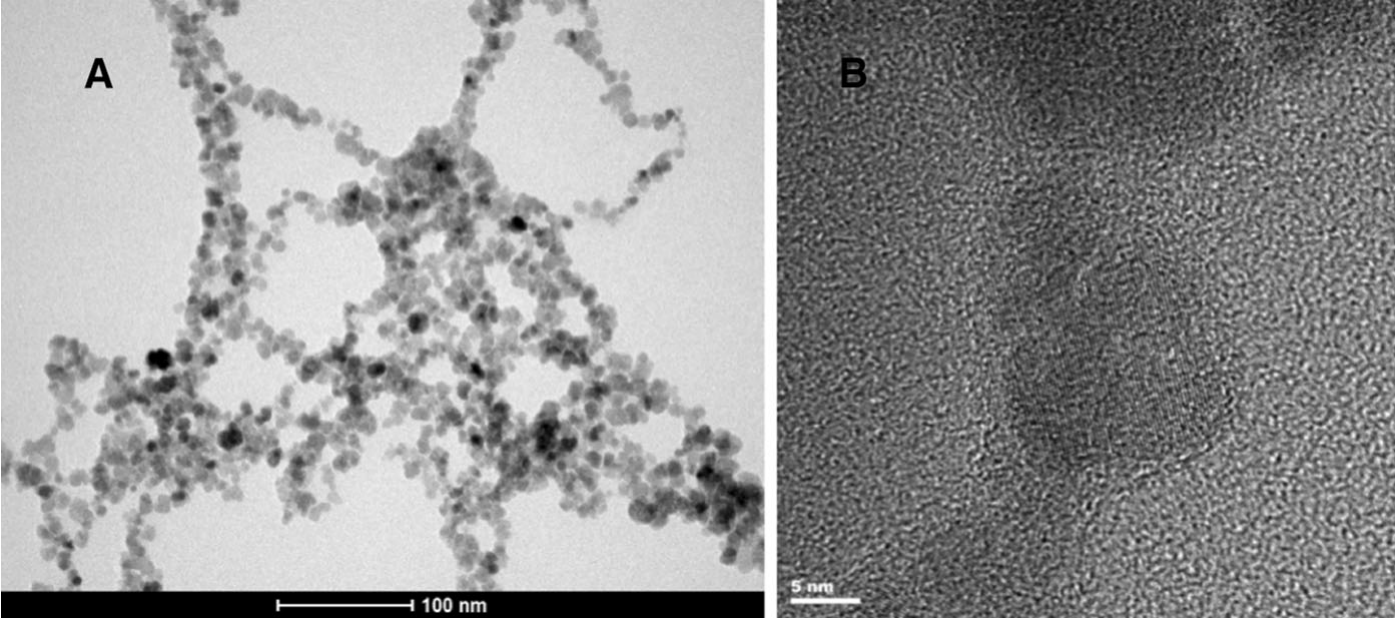
Bright-field TEM image (**a**) and HRTEM image (**b**) of SPION-Gd (electrostatic)

The shape and size of the nanoparticle core (for SPION-Gd (electrostatic)) were observed by the TEM imaging. The pictures reveal the crystalline structure of the material. Unfortunately, it was not possible to directly observe the presence of gadolinium on the surface of the nanoparticles due to too low sensitivity of the method. The obtained pictures (Fig. 4) for SPION-Gd (electrostatic) are consistent with the ones previously obtained by us for SPIONs (Szpak et al. 2013). Preliminary XRD studies revealed the presence of inverse spinel type structure and Mossbauer measurements indicated the presence of Fe^3+^ state of iron only, which corresponds to the fully oxidized magnetite, i.e., maghemite, γ-Fe_2_O_3_. The detailed discussion of that issue is beyond the scope of the current paper and it is going to be included in the following report.

#### X-ray fluorescence (XRF)

The results of XRF analysis of the SPIONs are collected in the Table 2. The values of gadolinium content are given as weight-percent or molar-percent, relatively to the sum of Fe and Gd content.

The above presented data show that the highest amount of gadolinium (11.5 wt%) was achieved for SPION-Gd (ACh-GdDTPA), a much smaller content of (3.8 wt%) was obtained for SPION-Gd (electrostatic), whereas the content of gadolinium in the SPION-Gd (GdCl_3_) was below the detection limit. As expected, there was no gadolinum in SPION-CCh nanoparticles (blank). The sensitivity limit for these measurements was about 0.1 %.

### Magnetic properties

#### Vibrating sample magnetometry (VSM)

**Fig. 5:**
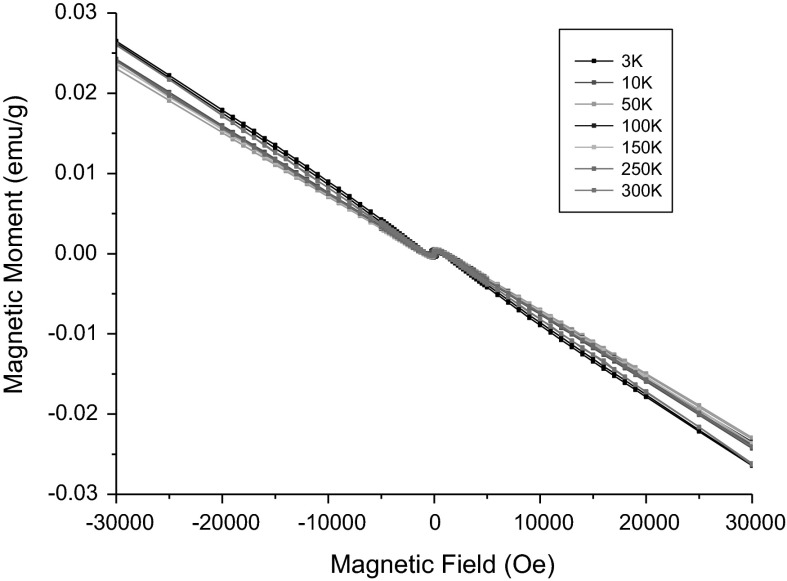
Hysteresis loops measured for the 10 ppm (Fe equivalent) water dispersion of the SPION-Gd (ACh-GdDTPA) nanoparticles at selected temperatures

The detailed magnetic studies were performed for SPIONs with the highest content of gadolinium. The hysteresis loops measured for the 10 ppm water dispersion of SPION-Gd (ACh-GdDTPA) at selected temperatures are presented in the Fig. 5.

**Fig. 6:**
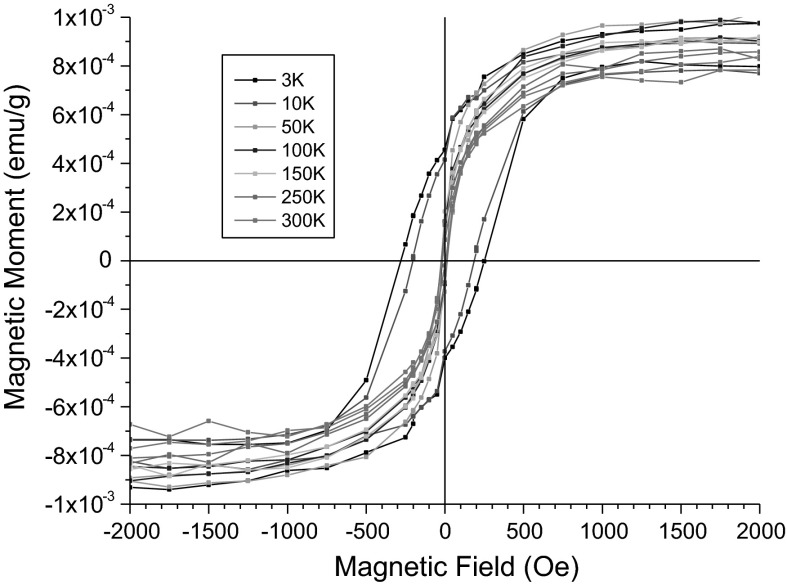
Hysteresis loops for SPION-Gd (ACh-GdDTPA) nanoparticles corrected for the diamagnetic contribution

These hysteresis loops reveal a substantial diamagnetic contribution. Data were corrected for this diamagnetic contribution by subtracting straight lines fitted to the linear parts of the plots at high field ranging from ±30 to ±80 kOe. Such corrected hysteresis loops are shown for the low field range in the Fig. 6.

The values of the coercive field Hc amounts to 290 Oe at 3 K and it decreases gradually to 190 Oe at 10 K, which means that the nanoparticles frozen in water exhibit a ferromagnetic or ferrimagnetic effect with a relatively high coercivity at liquid helium temperatures. Already at 50 K and also at higher temperatures, the coercive field is virtually zero, what indicates their superparamagnetic state. The obtained saturation magnetization of 75 emu/g related to the nanoparticle cores is very close to the value of 70 emu/g reported for maghemite nanoparticles of similar particle sizes (Berkowitz et al. 1968). It is worth noting that the magnetic saturation is obtained at the applied field ranging from 700 Oe under the temperature ranging from 50 to 250 K, i.e., in the solidified dispersion to 800 Oe at 300 K, in the liquid. A significant linear part of the magnetization curve with only a small nonlinear part, close to saturation, reveals a minor effect from magnetic inhomogeneities indicating a well ordered structure of the maghemite cores of the nanoparticles.

#### Relaxivity

**Fig. 7:**
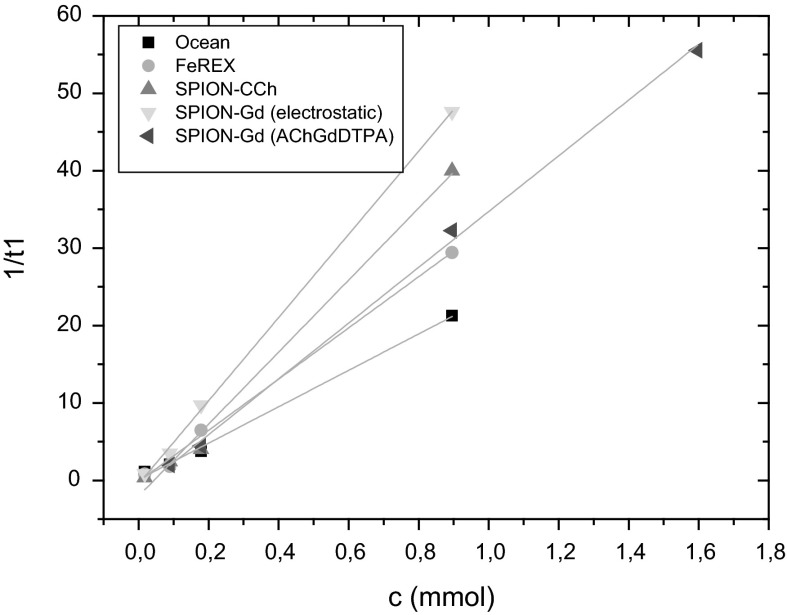
The dependence of the 1/T1 relaxivity values on the concentration of nanoparticles for prepared bimodal agents and commercial contrasts

**Fig. 8:**
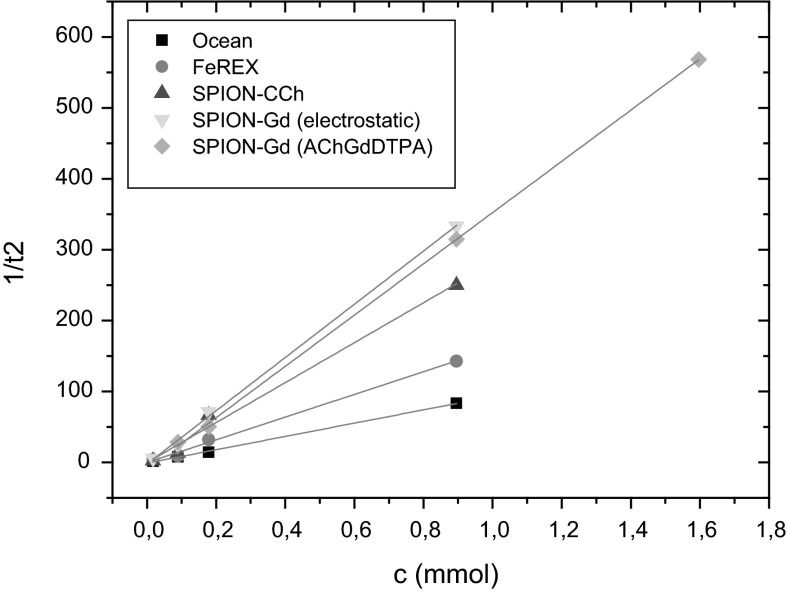
The dependence of the 1/T2 relaxivity values on the concentration of nanoparticles for prepared bimodal agents and commercial contrasts

In order to investigate potential usefulness of the prepared dual-mode MRI contrast agents relaxivity measurements were carried out. SPION-CCh and commercially used contrast agents: FeREX^TM^ and Ocean were also studied for a comparison. The resulting relaxivity values are presented in Table 3 and plotted at the Figs. 7 and 8.

**Table 3:** The relaxivity values obtained for prepared bimodal agents and commercially used contrasts

Sample name	r_1_ (mM^−1^ s^−1^)	r_2_ (mM^−1^ s^−1^)
SPION-Gd (ACh-GdDTPA)	36.0 ± 0.9	361.4 ± 4.3
SPION-Gd (electrostatic)	53.7 ± 1.1	375.5 ± 9.7
SPION-CCh	46.5 ± 2.8	282 ± 21
Ocean	23.5 ± 0.9	93.8 ± 1.9
FeREX^TM^	33.1 ± 1.3	160.1 ± 6.6

The 1/T_1_ and 1/T_2_ relaxivities show a linear dependence on the concentration of nanoparticles for the prepared bimodal agents as well as for commercially used contrasts. This means that contribution of individual nanoparticles to both relaxivities is additive, so that a possible agglomeration of the nanoparticles during the NMR measurement can be excluded.

As it was indicated in the introduction, the aim of our studies was to prepare the contrast agents with dual modality containing both: the superparamagnetic material (SPION) acting as negative contrast and paramagnetic gadolinium ion/complex working as a positive contrast. That novel material should shorten T_1_ and T_2_ relaxation times thus influencing r_1_ as well as r_2_ relaxivities. The calculated r_1_ and r_2_ values (see Table 3) show that the synthesized bimodal agents gave generally better results than for monomodal agent consisting of SPION-CCh only. In comparison to the commercially available contrasts, the relaxivities of the developed materials are significantly higher. In case of SPION-Gd (electrostatic), r_2_ value (375.5 mM^−1^ s^−1^) is about 2.5 times higher than that for FeREX^TM^ and almost four times higher than for Ocean (negative agents). The same applies for r_1_ value for SPION-Gd (electrostatic) (53.7 mM^−1^ s^−1^) that was found more than two times higher than for Ocean and slightly less for FeREX^TM^.

The presented results clearly indicate that the developed agents SPION-Gd (Ach-GdDTPA), SPION-Gd (electrostatic) are very promising candidates for MRI bimodal contrast enhancements. The SPION-Gd prepared based on the electrostatic interactions can be of special interest due to the high values for both r_1_ and r_2_ relaxivities.

## Conclusion

Three methods of preparing novel dual-mode nanostructural MRI contrast enhancement agents based on SPIONs and gadolinium were proposed. The obtained agents were characterized using several physicochemical techniques: FTIR, DLS, TEM, XRF, and their magnetic properties were evaluated by VSM and NMR.

SPION-Gd (ACh-GdDTPA) and SPION-Gd (electrostatic) approaches were very successful; the obtained materials have the outstanding relaxivity performance (high values of r_1_ and r_2_) considerably better than these for the commercially available contrast agents. In addition, the suspension of SPION-Gd (ACh-GdDTPA) exhibits very good long-term stability.

The methods developed allow for creating bimodal agents with different: gadolinium content, proximity of superparamagnetic core–gadolinium atoms, allowing penetration of polymer layer by water molecules, and various types of interactions (physical forces or chemical bond) therefore significantly influencing magnetic properties.

Additionally, the presented methods allow to change the type of polymer used to coat the iron oxide nanoparticles and precise control of the distance between magnetic core and gadolinium complex. This makes possible to create a very broad spectrum of materials for MRI but also various other practical applications. The obtained materials can be considered as highly effective contrast agents for low-field MRI, in particular at permanent magnet-based scanners.
